# Participant-Partners in Genetic Research: An Exome Study with Families of Children with Unexplained Medical Conditions

**DOI:** 10.2196/jopm.8958

**Published:** 2018-01-30

**Authors:** Sara Huston Katsanis, Mollie A Minear, Azita Sadeghpour, Heidi Cope, Yezmin Perilla, Robert Cook-Deegan, Duke Task Force For Neonatal Genomics, Nicholas Katsanis, Erica E Davis, Misha Angrist

**Affiliations:** ^1^ Duke Initiative for Science and Society Duke University Durham, NC United States; ^2^ Center for Human Disease Modeling Duke University Durham, NC United States; ^3^ School for the Future of Innovation in Society Arizona State University Tempe, AZ United States; ^4^ Duke University Medical Center Durham, NC United States; ^5^ Social Science Research Institute Duke University Durham, NC United States

**Keywords:** partnership, exome sequencing, genome sequencing, return of results, participant engagement

## Abstract

**Background:**

Unlike aggregate research on groups of participants with a particular disorder, genomic research on discrete families’ rare conditions could result in data of use to families, their healthcare, as well as generating knowledge on the human genome.

**Objective:**

In a study of families seeking to rule in/out genetic causes for their children’s medical conditions via exome sequencing, we solicited their views on the importance of genomic information. Our aim was to learn the interests of parents in seeking genomic research data and to gauge their responsiveness and engagement with the research team.

**Methods:**

At enrollment, we offered participants options in the consent form for receiving potentially clinically relevant research results. We also offered an option of being a “partner” versus a “traditional” participant; partners could be re-contacted for research and study activities. We invited adult partners to complete a pre-exome survey, attend annual family forums, and participate in other inter-family interaction opportunities.

**Results:**

Of the 385 adults enrolled, 79% opted for “partnership” with the research team. Nearly all (99.2%) participants opted to receive research results pertaining to their children’s primary conditions. A majority indicated the desire to receive additional clinically relevant outside the scope of their children’s conditions (92.7%) and an interest in non-clinically relevant genetic information (82.7%).

**Conclusions:**

Most participants chose partnership, including its rights and potential burdens; however, active engagement in study activities remained the exception. Not surprisingly, the overwhelming majority of participants—both partners and traditional—expected to receive all genetic information resulting from the research study.

## Introduction

### Researcher-Participant Partnerships: A Tough Walk to Walk?

Instantiation of reciprocal partnerships between researchers and participants has long been difficult to achieve, given: (1) historical norms of asymmetric researcher and participant relationships; (2) regulatory and policy disincentives to open communication (eg, Health Insurance Portability and Accountability Act [HIPAA] privacy provisions as applied to research participants; liability fears); and especially (3) practical and resource challenges to researchers [1]. Consequently, with few exceptions, one could argue that most unfettered big-data researcher-participant engagement has tended to happen in settings outside of academic medical centers (eg, Open Humans, PatientsLikeMe, Genetic Alliance) [2-5].

Partnership with patient participants in genomics-based research is embedded in the All of Us Research Program (formerly the Precision Medicine Initiative) and cited as an important component of open research communication that enables autonomy and choice in participation in long-term studies [6,7]. It was discussed as a salutary outcome in the 2015 Notice of Proposed Rulemaking that led to the reform of the Common Rule, the multi-agency framework that governs human participation in US research [8,9]. On a grassroots level, research participants have expressed both a strong willingness to share data derived from their samples and/or personal information and a desire to receive individual results from researchers [10].

Rule changes to HIPAA and the Clinical Laboratory Improvement Amendments of 1988 (CLIA) that took effect in 2014 suggest that research participants now have broad data access rights from any laboratory that behaves as a HIPAA-covered entity [11]. While some have regarded this development as “troubling,” we view it as an opportunity to begin to realize the aspirational notions of partnership expressed by All of Us, broadly by patient-centered research (eg, the Patient-Centered Outcomes Research Institute) and by participants themselves [12].

### Research-based Exome Sequencing and Functional Analysis in Undiagnosed Children: A Partnership Test Bed

The precipitous decline in the cost of whole-exome sequencing (WES) has made its use as a diagnostic approach increasingly commonplace [13-14]. Over the last several years, the power of WES to end diagnostic odysseys and, in some cases, to alter the course of clinical care, has been supported with an increasing number of examples [15-16].

Since 2011, our research group, the Task Force for Neonatal Genomics (TFNG), has received referrals from more than a dozen clinics within the Duke University Health System and elsewhere across the US for young children with congenital structural anomalies likely attributable to a genetic cause. Pediatric diagnostic challenges arise among many specialty clinics, with only a small proportion referred to medical genetics [17]. By engaging directly with the specialty clinics, in many cases prior to a genetics referral, we could begin exploring possible genetic causes and engage with families who might otherwise not have been considered for exome sequencing.

Here we describe the efforts to increase participant involvement in the research and subsequent effects; the enrollment processes will be described later in the paper. In general, if clinic referrals met our consensus inclusion criteria, we enrolled trios (or quads, etc, if more than one child were affected) of biological parents and children with undiagnosed conditions for research-based WES.

We exome-sequenced each individual (both biological parents and affected children), and filtered candidate alleles using published and in-house algorithms. Candidate causative alleles were confirmed by Sanger sequencing in all enrolled family members, then parsed further through literature searches and, when possible, modeled in zebrafish to test causality of suspect alleles [18-23]. Use of animal models in combination with WES is a flagstone of this research project and distinguishes our study from standard clinical exome sequencing. In some cases, families pursue clinical exome sequencing as well; in other cases, families have already received inconclusive clinical exome results and have enrolled in our research study for data reanalysis and the development of zebrafish assays. As of July 2016, we had enrolled 225 families, and returned results on 52 probands. The remaining families are currently in the analysis pipeline in our study.

We define partnership as a reciprocal exchange of information and communication to the benefit of all parties and participation as partners as an educative dividend of that exchange [24]. In our relevant research context – that is the investigation of a genetic cause to a child’s medical condition – the information exchange can be mutually beneficial to families of the child, researchers, and the health care providers caring for the child. In family-based research on a rare condition, the family-specific research data may be of personal utility (if not clinical utility) beyond use in aggregate research studies. From the outset of this project, our mission has been to engage with families seeking a genetic etiology for their child’s medical condition near the onset of their diagnostic odyssey, or at least from the point at which they are referred to Duke University Medical Center. For this reason, we designed our protocol to enable return of genetic information relevant to the child’s condition, whether it was conclusive or not. To guide the choices of families potentially wary of engagement with the clinicians and research team and to further this objective, we developed a “partnership” model for families.

**Figure 1:**
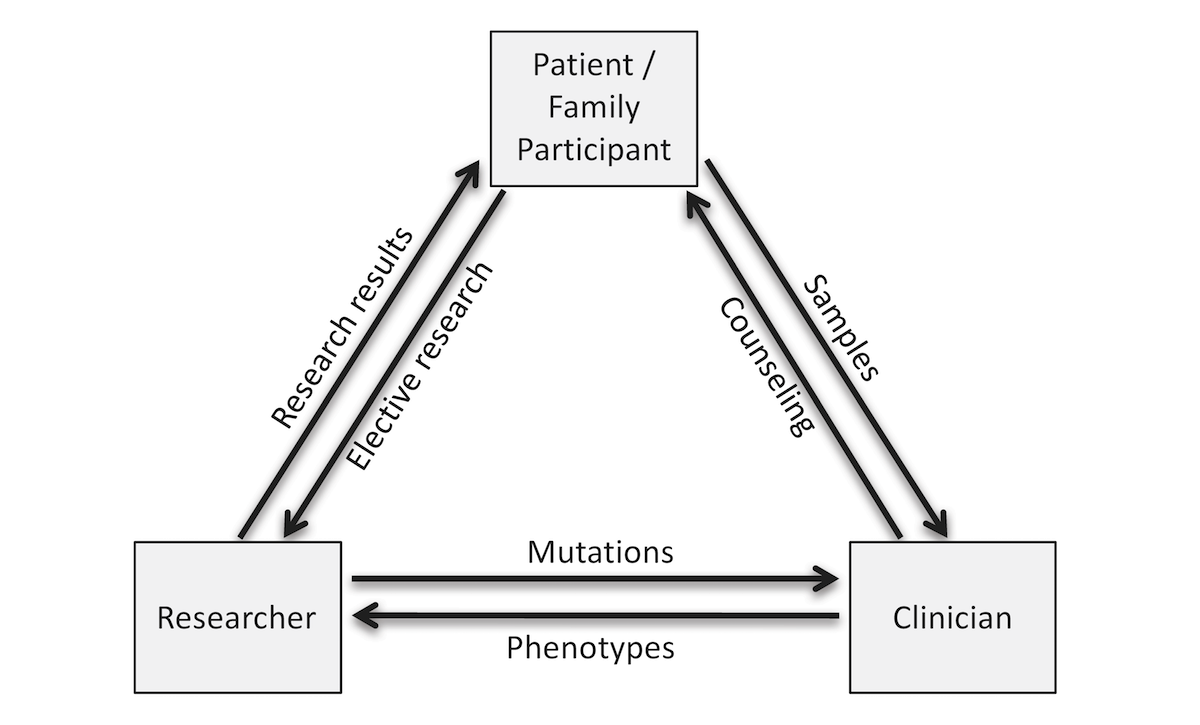
The partnership model of interchange of information fosters long-term prospective communication of phenotype, genetic risk, and interpretation of research results.

To that end, we developed three ways to engage family participants. First we developed a consent process that would allow partnership with families that would enable open communication between the research team and the participants, rather than relying upon the clinical referral team to transmit and receive information from individual participants (Figure 1). This partnership model is an option presented in the initial consent form. Second, we developed an online survey to gauge partner-participants’ pre-exome interests in aspects of the research, including return of research data. Third, we hosted three annual family forums with the aim of bringing participant families together with the researchers and clinicians managing their research exomes. Here we present our findings on partner participation among our exome research enrollees and their pre-exome interests in receiving genetic research data.

## Methods

### Participant Enrollment Process

All study materials and protocols were approved by the Duke University Health System Institutional Review Board (DUHS Pro00031066). Pediatric and prenatal probands were referred from clinics across Duke, primarily pediatric urology, neurology, cardiology, craniofacial, bone marrow transplant, maternal-fetal medicine, and neonatology. Consent materials were available in Spanish. Genetic counselors reviewed case medical histories and discussed the phenotypes of individual cases with referring clinicians. The study team prioritized enrollment of cases that: (1) were suspected to be genetic (eg, no known maternal confounding or environmental factors; actual or possible recurrence within the family); (2) had no prior molecular genetic diagnoses as determined by clinical genetic testing (eg, WES, panel sequencing, karyotyping, or array analysis); and (3) had phenotypes for which an anatomical surrogate could be modeled in zebrafish. Cases that did not meet pre-determined criteria were excluded by consensus or voted upon by study team members. Once the team decided to include a case, a health care professional known to the family introduced them to a research genetic counselor who then presented the study and consented willing family members, which, at minimum, included a trio of both parents and an affected child. The team required the availability of both biological parents to qualify for enrollment unless there were either multiple affected individuals or multiple generations of available family members.

### Consent Process

Genetic counselors described the scope of the study and the potential for obtaining exome-based research results, including variants directly relevant to the affected child’s condition, variants clinically relevant to the child or parent, and variants unrelated to the condition and/or not clinically relevant. The consent process included options for receiving directly relevant results, as well as clinically relevant results (ie, additional or “secondary” findings). The TFNG research protocol permitted return of clinically relevant results only; however, the consent form asked participants for their potential future interest in receiving results that were not clinically relevant, and a subsequent amendment to the protocol allowed for return of raw sequence data. Finally, the consent form provided an option to be a “partner” in the study or a “traditional participant” (relevant extracts of consent form available as supplementary information). Partners and researchers could communicate in an unfettered way via email; partner participants agreed to be re-contacted for surveys and invitations to Task Force events such as seminars and participant gatherings.

### Survey Development and Collection

Survey questions and design were developed in consultation with internal and external content experts, including genetic counselors, clinical geneticists, and genetic/genomic researchers. The survey questions (available as supplementary information) contained four main sections: (1) demographics (age; relationship to child; age of affected child); (2) experience in clinical or research-based approaches to identify an underlying cause of the child’s/children’s condition (length of diagnostic odyssey; number of specialists sought; length of time in research); (3) perspectives on research participation (rating scales); and (4) expectations for research (yes/no questions). In total, the survey consisted of 16 questions formatted as multiple-choice or nine-point sliding scales (ie, rate from 0-10). The survey was designed and distributed using Qualtrics software (Qualtrics, LLC, Provo, UT). Only adult parent participants who consented to be partners were eligible for the survey. Partner-participants who did not provide email addresses were excluded. The survey was conducted in English; participation was voluntary and anonymous.

Eligible participants were emailed an introduction to the survey, including a consent document and a link to the anonymous survey, within a month of the family’s samples having been collected and the WES pipeline having been initiated. A single reminder email was sent about a month after the initial introduction. Because responses were anonymous and not linked to family identifiers, in some cases both a father and mother may have responded to the survey. In some cases, individuals were re-consented on the exome protocol at a later date to expand their participation options, so those samples would have been undergoing analysis for longer than a month.

After survey data collection, we conducted quality-control checks to assure that value ranges and missing data codes were valid. Data were analyzed for possible sources of response bias including inspecting individual responses for extreme bias and evaluation of consistent data trends over time. Responses were summarized using frequency distributions. For sliding scales, the mean was used with standard deviations (SD) to demonstrate response clusters. Qualtrics and Microsoft Excel v.14.5.5 were used for all analyses. Sample sizes varied by question since participants were allowed to skip any questions they did not wish to answer. Descriptive analyses about expectations for and use of WES results were prepared for the entire survey population.

### Family Forum and Engagement

Partner-participants were invited to attend an annual family forum (“Duke Genomes Family Forum”, DGFF) in years 2013-2015. Partner participants who did not provide contact information were excluded. Also, only partner participants for whom DNA sequencing had commenced (meaning all samples had been received) were included. Each attending family was offered $100 to offset travel and/or childcare expenses for each event; most participant families were local/regional. On-site special-needs caregivers were provided to enable inclusion of the children. The family forums were daylong conferences with activities, presentations, and social opportunities for families to meet one another and interact with attending researchers and clinicians. The agenda for each event included a mix of social events and presentations by researchers, clinicians, and participants. Participants attending the 2014 and 2015 DGFF events were invited to be interviewed for an independent documentary co-produced by study team members [25]. All partner participants were invited to a private screening of the documentary in early 2016. Participants attending the 2015 DGFF were invited to form a participant advisory board for the project.

## Results

### Participant Families

Between January 2012 and July 2016 (55 months), we screened 1,256 cases, ultimately enrolling a total of 225 families. Until June 2013, all families were enrolled on a genetics protocol (DUHS Pro00022846) that was not specific to exome or genome sequencing. The majority of participants thereafter enrolled on the more comprehensive exome and return-of-results protocol described herein (DUHS Pro00031066). Many of the participants consented originally on the former protocol were re-consented onto the exome protocol prior to return of results. Of the 225 consented families, 193 families (385 adults) were initially or eventually consented on the exome protocol. Self-reported race and ethnicity indicate that of the 450 adult parents (225 families), 106 (23.6%) parents were non-white, including 51 (11.2%) African or African American parents, while 51 (11.2%) parents had Hispanic ethnicity. Note that while 225 families enrolled in the study under the original protocol, not all families or family members re-consented to the exome protocol, leaving 385 adult consents on the exome protocol.

### Consent Options

Of the 385 adults who enrolled on the exome protocol, 303 (78.7%) opted for “partnership” with the research team, while 82 (21.3%) opted for “traditional” participation. Of the 385 participants completing the exome consent form, four of the consent forms were blank for the options regarding return of direct results, indirect results, and future interest in non-clinical results, leaving 381 participants. No statistical significance was seen based on sex or race/ethnicity of participants. Nearly all adult participants (378/381; 99.2%) opted to receive results directly related to their children’s conditions. The three who chose not to receive results were “traditional” participants. Most participants (353/381; 92.7%) also opted to receive additional clinically relevant results unrelated to their children’s conditions. No statistical differences in choices were noted between partners who opted not to receive clinically relevant results and traditional participants. The majority of participants (315/381; 82.7%) also expressed an interest in receiving their own non-clinical results. Partners were somewhat more likely than traditional participants to elect this option, but this difference was not significant (*p*=0.08). No significant differences were observed between mothers versus fathers in selecting these options; the numbers were too small to parse by other demographic criteria.

### Survey Responses

The 303 partner participants were screened for eligibility for the survey. Excluded from this group were four participants pending consents or samples from one or both of the biological parents, and 32 participants who declined to provide email addresses. This left 267 eligible participants. Of these, 103 responded to the pre-exome survey (a 38.6% response rate) and 95 completed all questions in the survey (92.2% completion rate).

Of the respondents, the majority (80%) had a child under 10 with a condition that led to their interest in enrolling in the study. More mothers (78/103; 75.7%) responded to the survey than fathers (25/103; 24.3%; *p*<0.001). Despite the relatively young ages of the children, almost a third (n=103; 29.6%;) reported that they had been seeking a diagnosis for their child for more than five years; 19.2% among those with children under 10 (n=82). In their searches for diagnoses, parents reported consultation with as few as one specialist (13/97; 13.4%) to more than 20 specialists (11/97; 11.3%). Overall respondents consulted an average of 6.7 specialists (*SD*=6.19).

**Figure 2:**
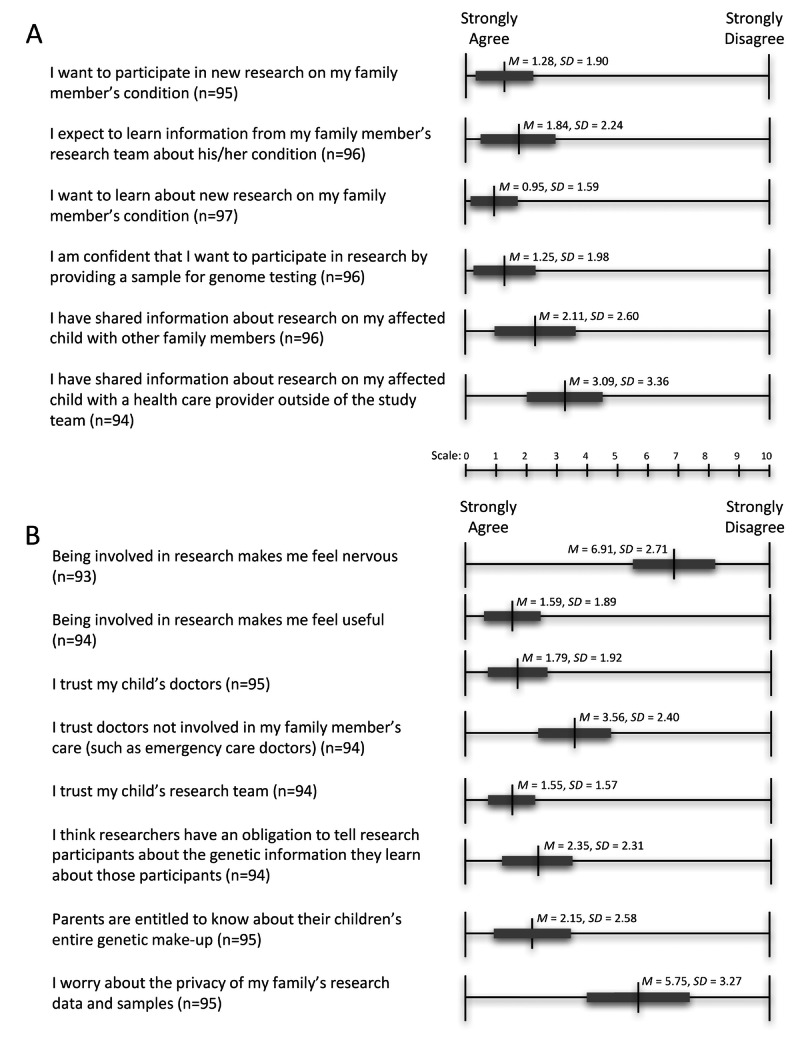
Participants were asked to scale from 1-10, with 1 being “strongly agree” and 10 being “strongly disagree,” their (A) reasons for participating in the research project, plans to share research information, and (B) feelings about participating in research. The vertical line represents the mean (M) and the thick gray line the standard of deviation (SD).

On the scale responses, participants indicated that they expected to learn information about their children’s conditions through the research study and showed a strong desire to participate in research (Figure 2). Participants also indicated that they had shared information about the research with both their family members and health care providers outside of the study team. Respondents indicated strong levels of trust in the study team and their children’s doctors, but less trust of doctors not directly involved in the care of their children. Participants indicated that researchers had an obligation to tell participants about the genetic information they learned from the research and that parents were entitled to access their children’s genetic information. Respondents were split as to whether research made them nervous (*M*=6.88; *SD*=2.78) and if they were worried about the privacy of research data and samples (*M*=5.88; *SD*=3.38).

**Figure 3:**
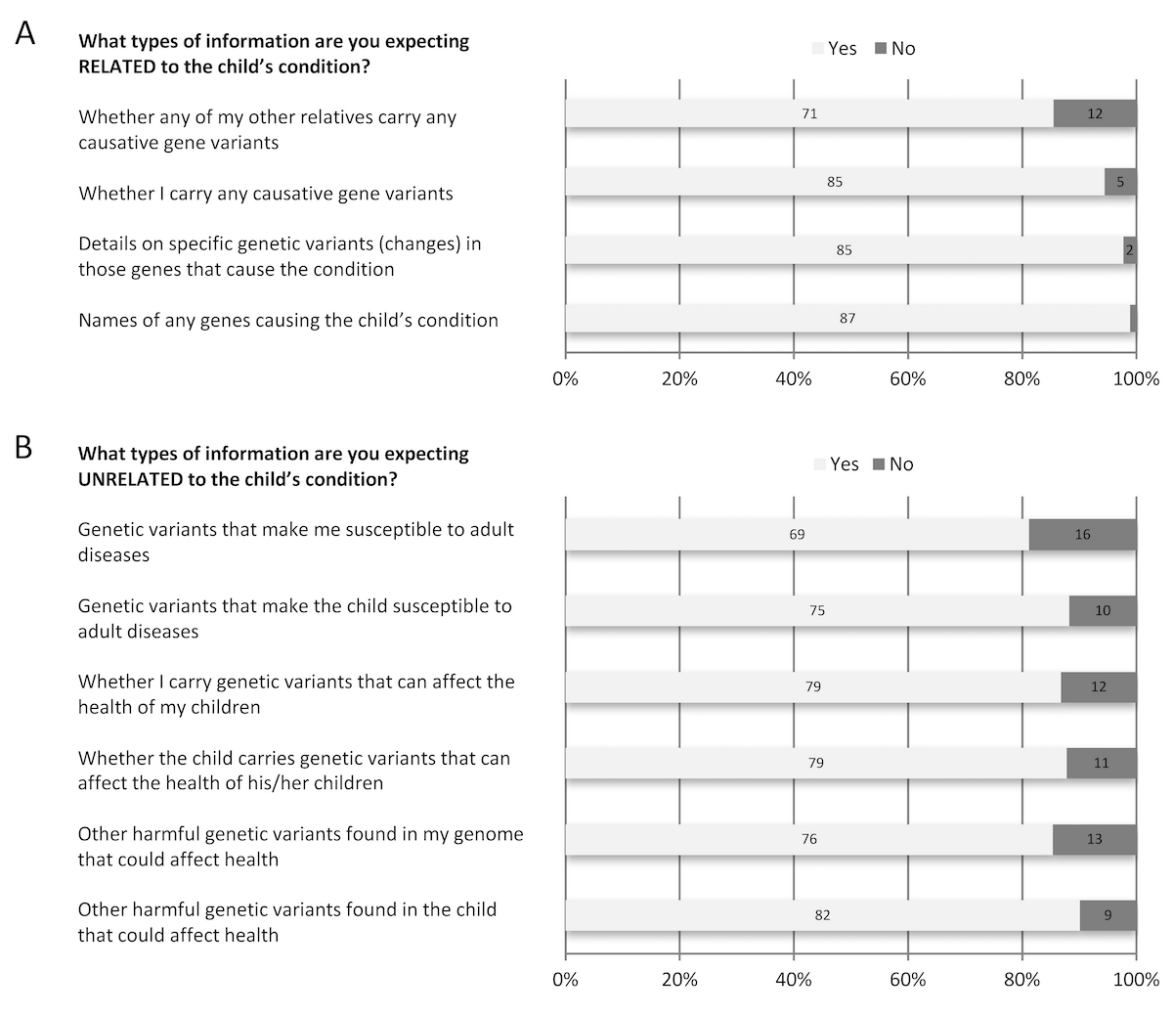
Participants were asked their expectations for receiving information (A) related to their child’s condition and (B) unrelated to their child’s condition.

**Figure 4:**
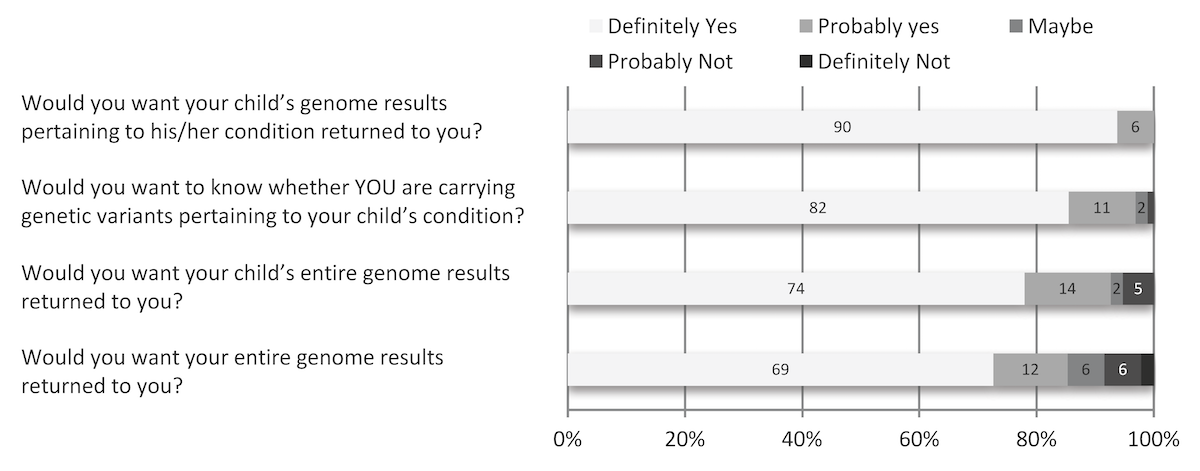
Participants were asked their desires for receiving information related to their child’s condition and for receiving their entire genome results.<.

Survey respondents overwhelmingly indicated expectations for receiving genetic information related to the causes of their children’s conditions, but also expected to get genetic information unrelated to their children’s conditions, including susceptibility to adult diseases for themselves (81.2%) and their children (88.2%; Figure 3). All survey respondents indicated a desire to receive genome results pertaining to their children’s conditions (Figure 4). Respondents also indicated a strong desire to know their own carrier status (96.9%) for autosomal recessive disorders, to receive their children’s exome data (92.6%), and to receive their own exome data (85.3%).

### Family Forum Participation and Project Engagement

Family forums were held during three consecutive summers from 2013-2015, with invitations extended to partner families. Each year a greater number of families qualified to attend as enrollment in the project increased. Invitations were sent to 70, 159, and 191 partner participants for each year, respectively. Absolute attendance remained fairly constant each year but declined proportionately as the overall number of participants grew: 21/70 (30%) in 2013; 27/159 (17%) in 2014; and 27/191 (14.1%) in 2015. Ten families were interviewed for the independent documentary; nine of the families were ultimately featured after one family declined continuing participation. The documentary was released to the public in 2017; the effect of the documentary on participants will be assessed in the future. In response to the study team’s suggestion of developing a participant advisory board, two families briefly considered the possibility but no further steps have been taken outside of the study team’s coordination.

## Discussion

We found that most adult research participants in a study of children with undiagnosed genetic diseases were strongly interested in obtaining genetic information about their children’s conditions and about themselves. In this respect our findings are in line with previous work on return of results in families undergoing WES in search of diagnoses [26-29]. In addition, adult participants in the present study were generally highly trustful of the biomedical research enterprise, not anxious about research participation, and relatively unconcerned about the prospect of their privacy being compromised (Figure 2).

Presenting the participants with the option of “partnership” at the onset of the study enabled us to conduct the survey and to engage with participants via other opportunities such as an annual family forum and seminars. We could not assess the reasons to decline partnership, as our protocol precluded approaching traditional participants for additional research. As the partner families receive research results, we continue to invite them to Task Force events and to solicit their interest in ongoing survey research. The “traditional participant” option, which was chosen by 20% of the cohort, allowed participants to receive research results but to decline deeper engagement (and associated time commitments), including our surveys.

Families encounter numerous specialists and expert opinions and may be enrolled in numerous research studies. Most families in our study have been seeking a diagnosis since the birth of their child; 11% had seen more than 20 specialists. Until a genetic etiology is determined, they often have few opportunities to engage with other families experiencing similar social and medical challenges. The overarching objective of our research study is to understand human genetic variation on a broad scale; but of course, this is not mutually exclusive with individual families learning something from their unique situations and their own particular genetic variants. We have tried to offer families opportunities through the family forums and other gatherings to learn about how the research is done, why it can take a long time, to meet other families seeking a genetic etiology for their children’s medical conditions, and to provide feedback to researchers and clinicians on their experiences and expectations. We co-produced an independent documentary film that followed families’ experiences in seeking diagnoses, enrolling/participating in our study, and negotiating the daily challenges of living with special-needs children.

We see partnership -- that is, open exchange of genetic research information and shared decision making -- as the most equitable framework for large-scale genomic studies and the one with the highest upside for researchers, clinicians and patients [30]. However, partnership comes at a high price and with significant challenges. The effort of a research team to engage individually with participants is significant, both in expense and time. The long turnaround time associated with genome sequencing, analysis, and modeling specific variants in zebrafish limits the pace with which we have been able to return final results, which frustrates clinicians, participant families and researchers alike. And obviously, families raising children with special needs have priorities that start with care of their children; engagement in research beyond provision of a genetic sample is likely to be of interest only when they believe they may realize some clinical and/or personal benefits [31]. Many families were unresponsive to invitations to the family forums, even with a small financial incentive for participation (though it is possible the incentive was insufficient). The families attending the family forums reported satisfaction with their experience. Eight participants attending the 2015 forum completed a post-family forum satisfaction questionnaire and no significant negative experiences were reported. For example, one participant commented:

It was really nice to be able to meet other families who are part of the study. It makes the study feel like it is more than just a study with ‘subjects.’ We also enjoyed being able to learn more about the study/research beyond our family.

However, the opportunity to meet other families negotiating similar circumstances, ongoing leadership among partner families for future community efforts has not coalesced. Attendance at the annual forums did not grow with the pace of the program and families have not come together to develop a participant advisory board that might influence the direction of the research and the institution’s approach to families with undiagnosed children.

Moreover, while the survey indicated a strong desire for receiving personal genetic data, at our institution the mechanisms and policies to enable research data sharing with participants continue to lag behind some other initiatives (eg, Geisinger; MyGene2). The inability to meet participant expectations can create frustration among participants who want information and among researchers who are reticent to provide incomplete and potentially uncertain data.

There were several limitations to our study. They include nonrandom ascertainment, ie, referring physicians were apt to be part of the Duke Health System and thus known to and trusted by participating families. In addition, given the rhetoric of partnership present in recruitment and online materials, our sample may well have been subject to a self-selection bias. Moreover, we did not include families who read the consent but ultimately chose not to enroll; their decision could easily have been influenced by privacy concerns—clearly this is a subject deserving of attention in subsequent research.

Diagnostic exome sequencing is less than a decade old. Remarkable progress has been made and the reference databases and number of sequenced exomes have grown exponentially [32-34]. At the same time, survey data have made it clear that genomic research participants expect to receive individual results [7,10,35,36]. Meanwhile, participants in the emerging biorights movement are refusing to contribute samples without assurances that they will: (1) be financially compensated; (2) receive relevant individual medical research information; and/or (3) be able to exercise some measure of control over the fate of their samples and data [37]. Moreover, the US Department of Health and Human Services Office for Civil Rights’ interpretation of the recent changes to CLIA and HIPAA suggests that research participants whose sequencing/genotyping was done in a HIPAA-covered lab have broad access rights not only to final interpreted test reports, but to all underlying genomic data that is traceable to them [38,39]. Thus, the partnership turn is now not only a moral and popular one, but a legal one.

That said, the ways in which we negotiate data access and participant expectations are a work in progress. Our hope is that the frank and forward-looking commitment of the National Institutes of Health to share individual results with large numbers of participants [40] will lead to: (1) the construction of a robust infrastructure for sharing genomic data and engaging with research participants; (2) active formation of support networks among other families living similar experiences involving genetic disease and uncertainty; and (3) a pervasive change in culture, that is, a day when information asymmetry is supplanted by true partnership.

## Abbreviations

CLIA: Clinical Laboratory Improvement Amendments
DGFF: Duke Genomes Family Forum
HIPAA: Health Insurance Portability and Accountability Act
PMI: Precision Medicine Initiative
SD: standard deviation
TFNG: Task Force for Neonatal Genomics
WES: whole exome sequencing
